# Medicinal wild plants used by the Mongol herdsmen in Bairin Area of Inner Mongolia and its comparative study between TMM and TCM

**DOI:** 10.1186/s13002-019-0300-9

**Published:** 2019-07-03

**Authors:** Wurchaih, Huar, Menggenqiqig, Khasbagan

**Affiliations:** College of Life Science and Technology, Inner Mongolia Normal University, Hohhot, 010022 People’s Republic of China

**Keywords:** Mongol herdsmen, Bairin Area, Wild plants, Folk medicine, TMM, TCM

## Abstract

**Background:**

Ethnobotanical studies on folk medicinal plants used by Mongol herdsmen have been conducted in some areas of Inner Mongolia and Xinjiang, China. However, ethnobotanical findings are preliminary and not comprehensive. Mongolian medicinal botanical knowledge has been gradually decreasing. One of the most important reasons is that Mongolian traditional medicine has become an alternative medicine in pasturing areas of China. Collection and analysis of Mongolian folk medicinal botanical knowledge have become extremely important.

**Methods:**

From 2008 to 2014, the authors have been to Bairin Right Banner seven times, and from 2016 to 2018, have been to Bairin Left Banner five times. Fieldwork was carried out in 18 villages, and 136 local Mongol herdsmen were interviewed. The methods of free-listing and open-ended questionnaires were used in field survey. Ethnobotanical interview and voucher specimen collections were organized in two ways: local plant specimens were collected beforehand and then interviews were organized; local Mongol herdsmen were invited to the field and were interviewed while collecting voucher specimens. Mongolian was used as the working language, and findings were recorded in Mongolian. Scientific names of plants were confirmed through collection and identification of voucher specimens.

**Results:**

Among the collected medicinal wild plants, 40 species are used by local Mongol herdsmen. Twenty-six species of folk medicinal plants have been recorded in the literature in the field of Traditional Mongolian Medicine (TMM), and 38 species have been recorded in the field of Traditional Chinese Medicine (TCM). The parts that have medicinal value include roots, whole plant, aerial parts, leaves, fruit, seeds, branches, bulb skin, and stem. For medicinal efficacy, among the collected medicinal plants, 8 species should be fresh. Thirteen species must be dried, and another 16 species can be fresh or dried. After a simple process of sorting and washing, local people soak, pulverize, and mash the medicinal plants. Nineteen species were externally used medicine, and 17 species were internally used medicine (taken orally). Generally, only one or two functions of folk medicine and indications associated with it were provided by local Mongol herdsmen. However, the functions of TMM and TCM and their indications show diversity and have systematic characteristics. More functions of TMM and TCM and their indications have been recorded. In the paper, we also discuss the correspondence between one or two functions of folk medicine with one or two functions of TMM and TCM.

**Conclusion:**

Not many medicinal wild plant species are used by Mongol herdsmen in the Bairin Area. Fourteen species have not been recorded in the literature of TMM, and 2 species have not been recorded in the literature of TCM. Folk knowledge can provide a certain reference value for searching for new medicinal wild plant species. On the whole, fresh plants are commonly used by Mongol herdsmen in the Bairin Area; boiling is the most common preparation method. Most of the crude materials can be used alone. The externally used medicinal parts are more common than those taken orally. A folk medicinal bath may be regarded as a compound drug mixed with two to five species of plant materials. The local Mongol herdsmen fully understand the function of folk medicines and their indications.

## Background

Traditional medicine is the sum total of the knowledge, skills, and practices based on the theories, beliefs, and experiences indigenous to different cultures, whether explicable or not, used in the maintenance of health as well as in the prevention, diagnosis, improvement, or treatment of physical and mental illness [1]. Traditional medicine has been used by the majority of the world population for thousands of years. The World Health Organization (WHO) reported that an estimated 80% of the population in developing countries depend on traditionally used medicinal plants for their primary health care [2]. The investigation of medicinal plants used by indigenous people is one of the most primary human concerns and has been practiced in China [3–7] and some other countries [8–19]. For example, the field investigation has documented traditional knowledge of the medicinal plants and their preparation methods and indications, used by the Yi ethnic group in Chuxiong, central Yunnan Province, Southwest China [3]; the study of medicinal plant resources and medicinal general patterns used of Caiçara living on the Atlantic Forest coast of Brazil also proposed important recommendations for resource conservation [9]; the survey interview of ethnomedicinal knowledge of the treatment of skin diseases by the medicinal plants used by people in the northeastern part of KwaZulu-Natal Province, South Africa [11], etc.

Traditional Mongolian Medicine (TMM), like Traditional Chinese Medicine (TCM), is one of the most important ethnic medicines with systematic theories. TMM has been developed over thousands of years among Mongolian people. TMM is based on Mongolian Folk Medicine (MFM). TMM has inherited many shamanistic practices with medical theories, techniques, and medications of Traditional Tibetan Medicine. The Mongolian medical system also has drawn on some aspects of other oriental medicines such as Ayurveda and Chinese medicine [20].

Today, TMM is an officially recognized medicine in China and the medicinal knowledge is mastered by the doctors who know about TMM. It is mainly rooted in medicinal literature, the regular hospital, medical colleges, and research institutes. However, MFM exists in the folk community and the knowledge is mastered by indigenous people such as herdsmen or peasants who have no formal educational experience in medicinal schools. Indigenous knowledge and herbal practices have not been written about in the medicinal books. In this case, MFM can be called Mongolian herbal medicine, which uses single or mixed plants to prevent or treat certain ailments or illnesses. The authors think that MFM and TMM are different stages of development of Mongolian medicine. MFM offers the possibility to contribute to and improve the system of TMM through in-depth study and medicinal practices [21].

Although TMM is based on MFM, the traditional knowledge of and the medicinal experience with medicinal plants used by Mongol herdsmen have not been given much importance in investigations and inventories made by researchers in the TMM field. They regularly rely on ancient books and records of Mongolian medicine, but not folk medicinal knowledge. Under these circumstances, in recent years, researchers in the ethnobotanical field have started to attach more importance to the folk knowledge on medicinal wild plants used by Mongol herdsmen.

Ethnobotanical studies on folk medicinal plants of Mongol herdsmen have been conducted in some areas of Inner Mongolia [22–27] and Xinjiang [28]. However, ethnobotanical findings are preliminary and less systematic. Mongolian traditional medicinal botanical knowledge has been gradually decreasing. One of the reasons is that western medicine was becoming more dominant in pasturing areas of China. Based on ethnobotanical investigations conducted from 2008 to 2014 in Bairin Right Banner and from 2016 to 2018 in Bairin Left Banner, Inner Mongolia Autonomous Region, northeast China, this paper aims to document the folk medicinal plant species and their medicinal parts and functions. Moreover, it provides the indications for which they are used.

## Materials and methods

### Study area and ethnic group

**Fig. 1:**
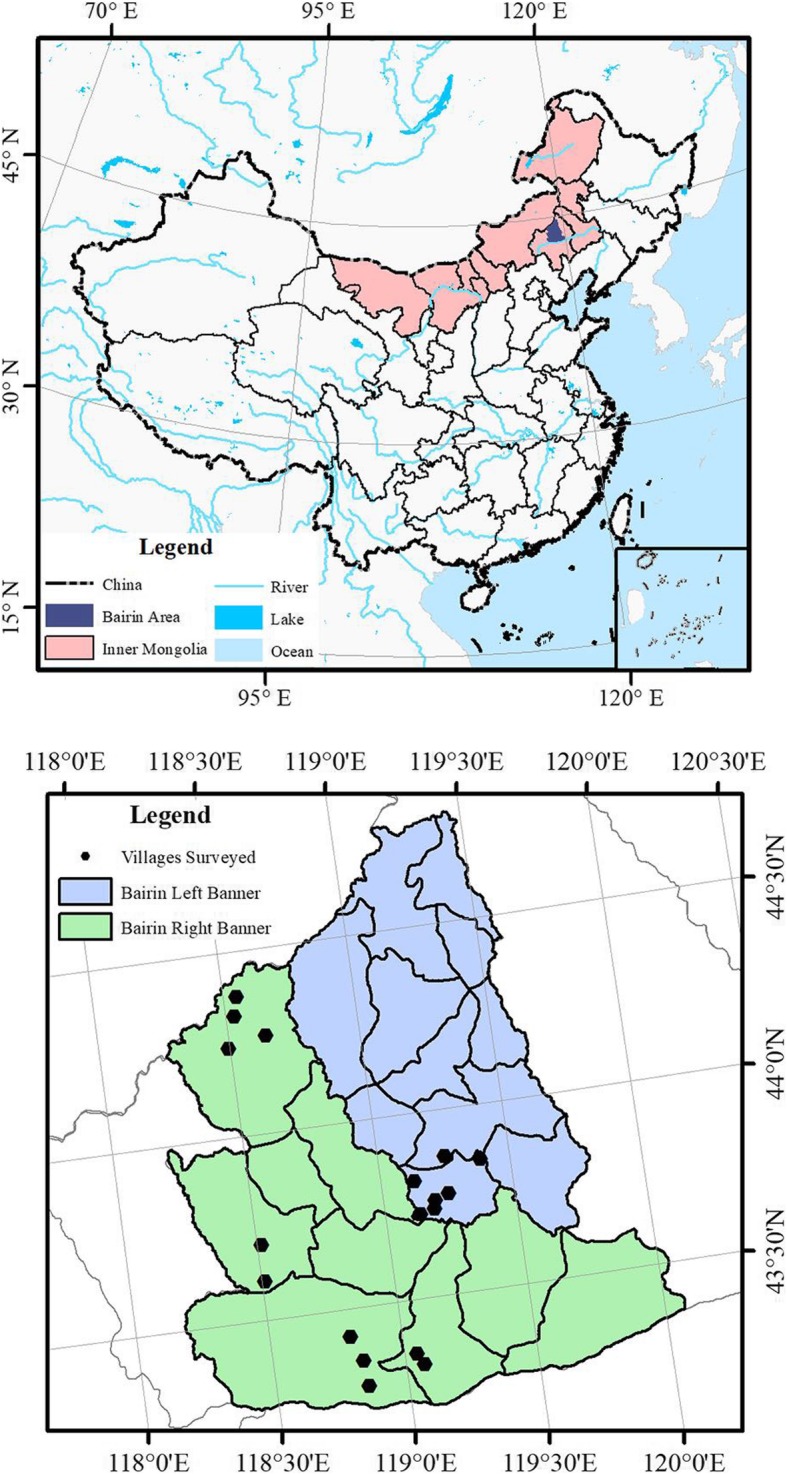
Study area and villages surveyed

Bairin Area is the collective name of Bairin Right Banner and Bairin Left Banner. The Bairin Area is located in northeastern Inner Mongolia, China. Bairin Right Banner, at 43° 12′–44° 28′ N and 118° 11′–120° 05″ E (Fig. 1), has a land area of 10,256 km^2^. The altitude range is from 400 to 1900 m [29]. Bairin Left Banner, at 43° 37′–44° 48′ N and 118°44′–119° 47″ E (Fig. 1), has a land area of 6635.5 km^2^. The average altitude is 600 m, and the highest peak is 1724 m [30]. The Bairin Area has a temperate zone continental climate, with an average annual temperature of 4.9 °C, and a mean rainfall of about 360 mm. The frost-free period in the area is about 125 days. The Bairin Area is situated in the transitional zone area which are mountains and hills of the Greater Hinggan Mountains transition to Yanshan Mountains. There are many geomorphic types: hills, sand, river lowlands, and lakes. Soil classifications in this zone are chestnut soil and brown loam. Other types of soil include eolian sandy soil, meadow soil, black soil, and gray forest soil. This area belongs to the typical steppe sub-band of the mesothermal steppe zone in the flora regional system of Inner Mongolia [31]. The vegetation form in the area is mainly forest, shrub, steppe, meadow, swamp, and aquatic vegetation.

According to “The Secret History of the Mongols,” Bodoncar, Genghis Khan’s tenth ancestor, seized a woman as a wife. After that, the woman bore a son named Baaridai. He became the ancestor of the Bairin tribe [32]. In 1648, during the Qing dynasty, the Bairin tribe formed a Banner in their occupied area and the name of the tribe was changed as the Bairin Banner. At the same time, the Bairin Area was divided into two banners, Bairin Right Banner and Bairin Left Banner. At present, more than 86,000 Mongol people are living in Bairin Right Banner and nearly 50,000 Mongol people are living in Bairin Left Banner. Most of the local Mongol people still engage in traditional animal husbandry dependent upon wild plant resources in this area and use the local wild plants for various traditional purposes.

### Methods

From 2008 to 2014, the authors went to Bairin Right Banner seven times. Fieldwork was organized in 11 villages including  (olʤi:t),  (luzi),  (ʧɑrstɑi),  (nɑrstɑi),  (bɑjɑnxɑn),  (ʃiwərtɑi),  (xotol),  (xɑnʊ:l),  (ɑrʃɑ:n),  (wangfengou), and  (bitu) (Fig. 1). Eighty-one local Mongol herdsmen were selected as key informants. From 2016 to 2018, the authors had been to Bairin Left Banner five times. Fieldwork was organized in seven villages including  (ʊlɑ:ngərəl),  (gɔlinɑil),  (ʧɑgɑ:ntɔhɔi),  (ɑrubʊlɑg),  (oworʤɔ:),  (bɑjɑnbʊlɑg), and  (xɑdɑngər) (Fig. 1).Fifty-five local Mongol herdsmen were selected as key informants. The names of the villages mentioned above are shown in traditional Mongolian, and the international phonetic sign is marked in parentheses [33]. The ethnobotanical interviewing methods included free-listing and open-ended questionnaires [34–39]. Ethnobotanical interviews and the collection of voucher specimens were carried out in two ways: the local plant specimens were collected and then the key informants were interviewed; the local Mongol herdsmen were invited to the field sites and were interviewed while collecting voucher specimens. Mongolian was used as the working language, and findings were recorded in Mongolian. Scientific names of plants were confirmed through the collection and identification of voucher specimens based on plant taxonomy.

## Results and discussion

### Ethnobotanical records

A total of 40 species of medicinal wild plants were used by the local Mongol herdsmen. Among them, 3 species belong to 3 different families of gymnosperm, and 37 species belong to 19 different families of angiosperm. Asteraceae, Rosaceae, Fabaceae, and Lamiaceae represent relatively more species (each with 9, 5, 4, and 4 species, respectively), accounting for 55% of the total species. Ethnobotanical data was recorded, as below, in the order of scientific names of species and family, life forms, local Mongol name, the used parts, methods, and indication or function (Table 1).

**Table 1:** Ethnobotanical inventory of the medicinal wild plants used by the Mongol herdsmen in Bairin Area

Scientific name	Family	Life forms	Local Mongol names^*^	Used parts	Methods	Indication and/or function	Specimen no.
*Adenophora wulingshanica* Maxim.	Campanulaceae	Perennial herbs	(sɔrlɔ), (xʊŋx ʧəʧəɡ)	Dried roots	Milling with *Clinopodium chinense* subsp. *grandiflorum* dry leaves, mixed powder to soak in boiled water.	Cough, clear away lung heat, reduce phlegm	CGHD-2017-074
*Allium condensatum* Turcz.	Liliaceae	Perennial herbs	(xɑdɑn sœŋɡ^j^ɔn), (nɔjɔn sœŋɡ^j^ɔn), (umxi: sœŋɡ^j^ɔn)	(a) Dried bulb skin, (b) fresh aerial parts	(a) Padded under the sole of the feet, (b) smell after calcining.	(a) Pregnant woman’s leg “ (bam)”; (b) toothache	BYBC-2008-028
*Artemisia argyi* Levl.	Asteraceae	Perennial herbs	(sʊix)	Fresh or dried whole plant	(a) Use soaked in boiled water or water-decocted solution to wash affected parts or bathing, (b) mixed with water-decocted solution of *Polygonum aviculare*’s fresh or dried roots, and bathing, (c) use soaked in boiled water or water-decocted solution apply on the forehead.	(a) Thermal spa treatment, dispel coldness, and disinfect; (b) dispel coldness; (c) headache	HG-2016-004
*Artemisia brachyloba* Franch.	Asteraceae	Subshrubs	(ʃɑrɑlʤ), (xɑdɑn ʃɑwɑɡ)	Dried whole plant	(a) Use water-decocted solution to wash affected parts or smoke the affected parts during calcining openly, (b) wash the affected part with water-decocted solution.	(a) Reduce swelling; (b) “ (ʃɑr)” disease (the body turns yellow)	BYB-2009-042
*Artemisia frigida* Willd.	Asteraceae	Perennial herbs	(æɡ^j^), (moŋɡon æɡ^j^)	Fresh or dried whole plant	Mixed with water-decocted solution of *Ephedra sinica*, *Rhododendron micranthum*, *Sanguisorba officinalis*, *Sabina vulgaris*, and bathing.	Joint pain	BYS-2009-010
*Cirsium maackii* Maxim.	Asteraceae	Perennial herbs	(ʊ:lɑ ows)	Fresh whole plant	The pulverized fresh whole plant is daubed on affected area.	Reduce swelling, stop hemorrhage	BYBC-2008-036
*Cirsium vlassovianum* Fisch.	Asteraceae	Perennial herbs	(ʊl ows)	Fresh leaves	Used with acupuncture therapy, roast silver needles on a white papery fresh leaves base and use.	Rheumatism and joint pain	CGHD-2017-023
*Clinopodium chinense* (Benth.) O. Ktze. subsp. *grandiflorum* (Maxim.)	Lamiaceae	Perennial herbs	(əlʤiɡən ʧix ows)	Dried leaves	Milling with *Adenophora trachelioides* dry roots, take powder to soak in boiled water.	Cough, heat-clearing, reduce phlegm	CGHD-2017-077
*Cuscuta chinensis* Lam.	Cuscutaceae	Annual parasitic herbs	(ʃɑr ɔrɔ:ŋɡɔ), (ʤəl ows)	Fresh or dried whole plant	Use water decocted solution to wash affected parts.	Trauma	BYBC-2007-023
*Ephedra sinica* Stapf	Ephedraceae	Small subshrubs	(ʤə:rɡən)	(a) Dried roots, (b) fresh aerial parts, (c) fresh or dried aerial parts, (d) fresh or dried aerial parts	(a) Smell the smoke of during calcining to ash, (b) take with water after roasted and ground, (c) decoct with water and take solution, (d) mixed with water-decocted solution of *Artemisia frigida*, *Rhododendron micranthum*, *Sanguisorba officinalis*, *Sabina vulgaris*, and bathing.	(a) Rhinitis, (b) relieves pain, (c) pneumonia, (d) joint pain	BYBC-2008-020
*Erodium stephanianum* Willd.	Geraniaceae	Annual herbs	(bʊɡʊn su:l ows), (bʊɡ ows)	Fresh roots	Eat fresh roots after low fire roast; neuropathy.	Body shiver	CGHD-2017-012
*Euphorbia esula* L.	Euphorbiaceae	Perennial herbs	(tɑrnʊ)	Fresh roots	Remove the root-bark and central hard parts, boiled with sheep bone soup and sun-drying, ground and add water, daubed on the infected part.	Trauma	BYBC-2007-028
*Glycyrrhiza uralensis* Fisch.	Fabaceae	Perennial herbs	(ʃixər ows), (bʊrʧɑɡ ows)	Fresh or dried roots	Soaked in cow milk and then decoct with water, take decoction.	Cough and vomit	BYBC-2008-092
*Incarvillea sinensis* Lam.	Bignoniaceae	Annual herbs	(sətɡu:r ows), (imɑn əwər ows)	Fresh or dried roots	(a) Gargled in the mouth with water decocted solution; (b) water-decocted solution for bathing.	(a) Toothache, (b) joint pain	BYBC-2008-047
*Iris dichotoma* Pall.	Iridaceae	Perennial herbs	(xɑiʧ ows), (bɔŋ ɡʊ:), (ɑɡʃirɡɑn)	Fresh roots	Remove the root bark, cut into pieces, and bitten between the teeth; the local herdsmen believed that the most efficacious time to collect the roots of this plant was on May 5 in the Chinese lunar calendar.	Toothache and gum swelling	BYBC-2009-003
*Leontopodium leontopodioides* (Willd.) Beauv.	Asteraceae	Perennial herbs	(moŋɡon æɡ^j^), (ʊ:lɑ ows)	Fresh leaves	Rolled into balls and packed into nostrils.	Nose hemorrhage	BYB-2009-062
*Oxytropis myriophylla* (Pall.) DC.	Fabaceae	Perennial herbs	(bʊrʧɑɡ ows)	Fresh whole plant	Mashed and daubed on the infected part.	Nose hemorrhage, trauma, reduce inflammations	BYBC-2009-007
*Pinus tabuliformis* Carr.	Pinaceae	Trees	(nɑrs)	Dried seeds	Eaten as nuts after frying.	Cough	BYBC-2009-040
*Plantago depressa* Willd.	Plantaginaceae	Annual herbs	(uxər urɡən)	Fresh or dried roots	Take water decocted solution.	Stomachache, diarrhea	CGHD-2017-03
*Polygonum aviculare* L.	Polygonaceae	Annual herbs	(bodonin su:l)	Fresh or dried roots	Mixed with water-decocted solution of *Artemisia argyi*’s fresh or dried whole plant, and bathing.	Dispel coldness	CGHD-2017-078
*Potentilla longifolia* Willd.ex Schlecht	Rosaceae	Perennial herbs	(ɑlɡɑlɑɡ tʊ:lɑin tɑŋnɑi)	Dried whole plant	Take water decocted solution.	Stomachache, enteritis	CGHD-2017-099
*Prunus humilis* Bunge (*Cerasus humilis* (Bunge) Sok.)	Rosaceae	Shrubs	(ʊlɑ:nɑ)	(a) Dried root, (b) fresh root, (c) fresh or dried roots	(a) Use water-decocted solution to wash affected parts or bathing; (b) remove the root bark, cut into pieces, and bitten between the teeth; (c) gargled in the mouth with water decocted solution.	(a) Children scabies, (b) toothache, (c) toothache	BYBC-2009-032
*Prunus sibirica* L. (*Armeniaca sibirica* (L.) Lam.)	Rosaceae	Trees	(xɑrɡɑn), (xə:rin ɡuils)	Dried kernel of seeds	Take water-decocted solution.	Cough, pneumonia, and tracheitis	BYBC-2007-008
*Pulsatilla chinensis* (Bunge) Regel.	Ranunculaceae	Perennial herbs	(ɡʊlɡʊi xʊɑr)	Dried aerial parts collected in the fall	Take water-decocted solution.	Body pains	BYBC-2010-007
*Pyrus ussuriensis* Maxim.	Rosaceae	Trees	(ʃɑr æl^j^ɑm), (xə:rin æl^j^ɑm)	Dried mature fruits	Soak in boiled water and take this water.	Baby’s cough	BYBC-2010-006
*Rhododendron micranthum* Turcz.	Ericaceae	Evergreen shrubs	(xɑrɑwɑr)	Fresh or dried branches	Mixed with water decocted solution of *Artemisia frigida*, *Ephedra sinica*, *Sanguisorba officinalis*, *Sabina vulgaris*, and bathing.	Joint pain	BYBC-2008-026
*Sabina vulgaris* Ant. (*Juniperus sabina* L.)	Cupressaceae	Shrubs	(xœn^j^n ɑrʧ)	Fresh or dried branches	Mixed with water-decocted solution of *Artemisia frigida*, *Ephedra sinica*, *Rhododendron micranthum*, *Sanguisorba officinalis*, and bathing. *Sabina vulgaris* is not native to Bairin Area; herdsmen got the raw materials of this plant from other places.	Joint pain	BYB-2009-173
*Sanguisorba officinalis* L.	Rosaceae	Perennial herbs	(sod ows), (sodon ʧɑi)	Fresh or dried roots	Mixed with water-decocted solution of *Artemisia frigida*, *Ephedra sinica*, *Rhododendron micranthum*, *Sabina vulgaris*, and bathing.	Joint pain	BYBC-2008-104
*Saposhnikovia divaricata* (Trucz.) Schischk.	Apiaceae	Perennial herbs	(ʃu:rɡən)	Dried roots	Take water-decocted solution.	Rheumatism and joint pain	CGHD-2017-102
*Scutellaria baicalensis* Georgi	Lamiaceae	Perennial herbs	(bɔr xʊnʧir)	Fresh or dried roots	Take water-decocted solution.	Clearing heat-fire, cough, detoxifying	CGHD-2018-036
*Scutellaria viscidula* Bunge	Lamiaceae	Perennial herbs	(ʃɑr xʊnʧir)	Fresh or dried roots	Take water-decocted solution.	Clearing heat-fire, cough, detoxifying	CGHD-2017-019
*Sonchus arvensis* L.	Asteraceae	Perennial herbs	(ɡɑʃʊ:n nɔɡɔ:)	Fresh or dried, whole plant	Eaten as vegetable for daily diet after washing or take soaked in boiled water.	Blood circulation, clearing heat-fire	HG-2016-010
*Sophora flavescens* Soland.	Fabaceae	Perennial herbs	(dɔɡlɔŋ ɔls), (dɔɡɔl ows)	Fresh or dried roots	Take water-decocted solution.	Heat-clearing and detoxifying	BYBC-2009-048,
*Sphaerophysa salsula* (Pall.) DC.	Fabaceae	Perennial herbs	(nɔxɔin bo:r), (xʊŋx ows), (pʊ:ʤiŋ ows)	Dried seeds	Water-decocted solution is used for bathing.	Back leg pain	BYBC-2009-005
*Stellera chamaejasme* L.	Thymelaceae	Perennial herbs	(dɑlɑn tu:ru:), (ʧudəŋ xʊɑr)	(a) Dried roots, (b) fresh or dried roots	(a) Roots are burned to ashes at a windproof place and mixed with alum, (b) take water decocted solution for foot soak.	(a) Diarrhea, (b) reduce swelling, joint pain	HG-2016-039
*Taraxacum mongolicum* Hand.-Mazz.	Asteraceae	Perennial herbs	(bɑɡwɑ:xai ʧəʧəɡ)	Fresh or dried whole plant	Take water decocted solution or soaked in boiled water.	Clearing heat-fire, hepatobiliary disease, fatty liver	HG-2016-038
*Thymus serpyllum* L. var. *asiaticus* Kitag.	Lamiaceae	Shrubs	(ʤɑ:r ows)	Dried aerial parts	Take water decocted solution.	Thermal spa treatment treatments, hemorrhoids	HG-2016-006
*Tribulus terrestris* L.	Zygophyllaceae	Annual herbs	(tomor ʤɑŋɡʊ:)	Dried mature fruits	Grind into powder after frying, take soaked in boiled water.	Renal edema	HG-2016-022
*Urtica cannabina* L.	Urticaceae	Perennial herbs	(xɑlɡɑi)	(a) Fresh whole plant, (b) fresh tender stem and leaves	(a) Eaten as vegetable after washing or water decocted solution is used for washing feet, (b) mashed and daubed on the infected part.	(a) Joint pain, (b) viper bites and analgesic effect	CGHD-2017-073
*Xanthium mongolicum* Kitag.	Asteraceae	Annual herbs	(xœn^j^n ʤɑŋɡʊ:)	Dried mature fruits	(a) Take oil daubed on the head after frying, (b) milling dried seed after frying, powder daubed on affected area.	(a) Children do not grow hair, (b) trauma	HG-2016-036

^*^The local Mongol names of medicinal plants in the table are shown in traditional Mongolian, and the international phonetic sign is marked in parentheses [33]

### Life form characteristics of folk medicinal plants

This area is a part of the temperate grassland of Eurasia. The characteristic of the temperate grassland is that grass is the dominant vegetation and trees and large shrubs are uncommon. The life forms of medicinal plants used by the local Mongol herdsmen are tree, shrub, subshrub, annual herb, perennial herb, and herbaceous vine. Among them, 24 species are perennial herbs and account for 60% of the total species (Table 2). Shown in Table 2, the local Mongol herdsmen frequently use herbaceous plants for medicine.

**Table 2:** Life forms of the plants

Parts used	Number of related species		Percentage	
Woody plants	9		22.5	
Tree		3		7.5
Shrub		4		10.0
Subshrub		2		5.0
Herbaceous plants	30		75.0	
Annual herb		6		15.0
Perennial herb		24		60.0
Vine	1		2.5	
Herbaceous vine		1		2.5
Total	40	40	100	100

### Species composition similarities compared with folk medicinal plants with TMM and TCM

According to the corresponding records about Chinese herbs and Mongolia medicine [40–43], 26 species of folk medicinal plants have been recorded in the literature of TMM, and 38 species have been recorded in the literature of Traditional Chinese Medicine (TCM). Two species*, Allium condensatum* and *Xanthium mongolicum*, have not been recorded in the relevant literature (Table 3).

**Table 3:** Folk medicinal plants recorded in TMM and TCM

Folk medicinal plants	TMM	TCM
*Adenophora wulingshanica*		+
*Allium condensatum*		
*Artemisia argyi*	+	+
*Artemisia brachyloba*	+	+
*Artemisia frigida*	+	+
*Cirsium maackii*		+
*Cirsium vlassovianum*		+
*Clinopodium chinense* subsp. *grandiflorum*		+
*Cuscuta chinensis*	+	+
*Ephedra sinica*	+	+
*Erodium stephanianum*	+	+
*Euphorbia esula*		+
*Glycyrrhiza uralensis*	+	+
*Incarvillea sinensis*	+	+
*Iris dichotoma*		+
*Leontopodium leontopodioides*	+	+
*Oxytropis myriophylla*	+	+
*Pinus tabuliformis*	+	+
*Plantago depressa*	+	+
*Polygonum aviculare*		+
*Potentilla longifolia*	+	+
*Prunus humilis*		+
*Prunus sibirica*	+	+
*Pulsatilla chinensis*	+	+
*Pyrus ussuriensis*	+	+
*Rhododendron micranthum*	+	+
*Sabina vulgaris*	+	+
*Sanguisorba officinalis*		+
*Saposhnikovia divaricate*		+
*Scutellaria baicalensis*	+	+
*Scutellaria viscidula*	+	+
*Sonchus arvensis*	+	+
*Sophora flavescens*	+	+
*Sphaerophysa salsula*		+
*Stellera chamaejasme*	+	+
*Taraxacum mongolicum*	+	+
*Thymus serpyllum* var. *asiaticus*		+
*Tribulus terrestris*	+	+
*Urtica cannabina*	+	+
*Xanthium mongolicum*		
Total	26	38

The similarity in selections of plant species between folk medicinal plants and TMM or TCM yielded a computational correspondence of 65% and 95%, which can be considered a high level of consistency between folk medicinal knowledge and TMM or TCM. Consistency between folk medicine and TMM shows the fact that the folk medicinal knowledge of Mongol herdsmen in the Bairin Area and TMM belong to the same medicinal system. Moreover, consistency between folk medicine and TCM shows knowledge exchange and cultural infiltration between the local Mongol people and Han Chinese. However, the similarity in medicinal plant species between folk medicinal plants and TCM is higher than the consistency between folk medicinal plants and TMM. How to understand this phenomenon? Why is it more similar with TCM? At present, the answer may be that TCM represents a broader range of medicinal practices sharing common theoretical concepts, has a historical tradition of over 2000 years, and includes various forms of herbal medicine with medicinal plant species which are richer than other species.

### The used parts of folk medicinal plants and their similarities of TMM and TCM

#### The used parts of folk medicinal plants

The used parts/product of medicinal plants include roots (16 species), bulb skin (1 species), whole plant (10 species), aerial parts (4 species), seeds (5 species), branches (2 species), leaves (4 species), and fruit (1 species). Two species of medicinal plants can be chosen for their two kinds of the used parts, and one species of medicinal plants can be chosen for its three kinds of the used parts, i.e., bulb skin and whole plant of *Allium condensatum*; root and aerial parts of *Ephedra sinica*; whole plant, tender stem, and leaves of *Urtica cannabina*. This is the reason why the number of related species is greater than the total of plant species (Table 4).

**Table 4:** The used medicinal parts/product part of the plants

Plant part	Number of related species	Percentage
Roots	16	36.36
Whole plant	10	22.73
Aerial part	4	9.09
Leaves	4	9.09
Fruit	3	6.82
Seeds	3	6.82
Branches	2	4.55
Bulb	1	2.27
Stem	1	2.27
Total	44	100

Among the plant parts used for medicinal purposes, roots (36.364%) are the most frequently used, and the aerial part (9.091%), if we count in the leaves (9.091%), branches (4.545%) and stem (2.273%), total of 25%, follows as second. However, the whole plant (22.727%) is the third most frequently used part. Overground parts, i.e., aerial parts, branches, leaves, stem, seeds, or fruits, of 16 species (tender stem and leaves of *Urtica cannabina*) are used for medicine. Underground parts of plants which include bulb skin and roots have 17 species related. Ten species of whole plant or whole plant is used for medicine.

#### Similarities in the used parts compared with TMM and TCM

Except *Allium condensatum* and *Xanthium mongolicum*, 38 species of folk medicinal plants are used in TMM or TCM (or both). In terms of the medicinal used parts, the folk medicinal used parts of seven species are the same among the three plants (*Scutellaria baicalensis*, *Scutellaria viscidula*, *Sonchus arvensis*, *Sophora flavescens*, *Stellera chamaejasme*, *Taraxacum mongolicum*, *Tribulus terrestris*). The folk medicinal used parts of another six species are the same with that of TCM (*Adenophora wulingshanica*, *Artemisia brachyloba*, *Cirsium maackii*, *Sanguisorba officinalis*, *Saposhnikovia divaricata*, *Thymus serpyllum* var. *asiaticus*). The folk medicinal used parts of two species are the same as that of TMM (*Potentilla longifolia*, *Pyrus ussuriensis*).

Among 17 species of the folk medicinal plants, 1 of the medicinal used parts is the same as TMM or TCM. For example, the whole plant is mostly plant parts of underground and their aboveground parts. For the *Ephedra sinica*, roots and aerial parts are used in folk medicine. However, in TMM, the herbaceous stem is used while the herbaceous stem and root are used in TCM. Among them, folk medicine and TCM are the same in using the root and herbaceous stem as the main part of aerial parts of *Ephedra sinica*. For the *Glycyrrhiza uralensis*, its roots and rhizome are used in TMM and TCM. However, in folk medicine, only the root is used. For the *Leontopodium leontopodioides*, in TMM and TCM, the aerial part is used, but only the leaves are used in folk medicine. For the *Prunus sibirica*, in TMM and TCM, the seed is used, but in folk medicine, only the kernel of the seed can be used. For the *Pinus tabuliformis*, in TMM, its nodular branch, leaves, cones, and pollen can be used, and the nodular branch, leaves, and cones can be used in TCM. But in folk medicine, only the seed is used. However, the seed may be contained in a cone. The used part of six species of folk medicinal plants is totally different in TMM or TCM. For example, the whole plant of *Cuscuta chinensis* is used in folk medicine, but only its seed is used in TMM and TCM. The root of *Prunus humilis* is used in folk medicine, but only the kernel of its seed is used in TCM (Table 5). Comparing TMM with TCM, the medicinally used parts of 18 species are totally the same.

**Table 5:** Comparison of the used parts/product used among folk medicinae, TMM and TCM

Folk medicinal plants	Folk medicine	TMM	TCM
*Adenophora wulingshanica*	Roots	–	Roots
*Artemisia argyi*	Whole plant	Leaves	Leaves
*Artemisia brachyloba*	Whole plant	Aerial parts	Whole plant
*Artemisia frigida*	Whole plant	Aerial parts	Aerial parts
*Cirsium maackii*	Whole plant	–	Whole plant
*Cirsium vlassovianum*	Leaves	–	Whole plant, roots
*Clinopodium chinense* subsp. *grandiflorum*	Leaves	–	Whole plant
*Cuscuta chinensis*	Whole plant	Seeds	Seeds
*Ephedra sinica*	Roots, aerial part	Herbaceous stems	Herbaceous stems and roots
*Erodium stephanianum*	Roots	Whole plant	Whole plant
*Euphorbia esula*	Roots	–	Whole plant
*Glycyrrhiza uralensis*	Roots	Roots and rhizome	Roots and rhizome
*Incarvillea sinensis*	Roots	Aerial parts	Aerial parts
*Iris dichotoma*	Roots	–	Rhizome or whole plant
*Leontopodium leontopodioides*	Leaves	Aerial parts	Aerial parts
*Oxytropis myriophylla*	Whole plant	Aerial parts	Aerial parts
*Pinus tabuliformis*	Seeds	Nodular branch, leaves, cones, pollens, resin	Nodular branch, leaves, cones
*Plantago depressa*	Roots	seeds	Seeds, whole plant
*Polygonum aviculare*	Roots	–	Aerial parts
*Potentilla longifolia*	Whole plant	Whole plant	Whole plant, roots
*Prunus humilis*	Roots	–	Kernel of seeds
*Prunus sibirica*	Kernel of seeds	Seeds	Seeds
*Pulsatilla chinensis*	Aerial parts	Whole plant	Roots
*Pyrus ussuriensis*	Fruits	Fruits	Fruits and leaves
*Rhododendron micranthum*	Branches	Leaves or flowering branches	Branches and leaves, flowers
*Sabina vulgaris*	Branches	Branches and leaves	Branches and leaves
*Sanguisorba officinalis*	Roots	–	Roots
*Saposhnikovia divaricata*	Roots	–	Roots
*Scutellaria baicalensis*	Roots	Roots	Roots
*Scutellaria viscidula*	Roots	Roots	Roots
*Sonchus arvensis*	Whole plant	Whole plant	Whole plant
*Sophora flavescens*	Roots	Roots	Roots
*Sphaerophysa salsula*	Seeds	–	Whole plant and fruits
*Stellera chamaejasme*	Roots	Roots	Roots
*Taraxacum mongolicum*	Whole plant	Whole plant	Whole plant
*Thymus serpyllum* var. *asiaticus*	Aerial parts	–	Aerial parts
*Tribulus terrestris*	Fruits	Fruits	Fruits
*Urtica cannabina*	Whole plant, leaves, stem	Whole plant	Whole plant

In respect to the medicinal parts used between MFM and TMM or TCM, complete and partial similarity indicates an interrelationship among MFM, TMM, and TCM. The differences in the medicinally used parts between MFM and TMM or TCM can provide information of medicinal materials for further study.

### Preparation methods of folk medicine and their similarities between TMM and TCM

#### Fresh and dried medicine

Folk medicines can be classified as fresh medicine and dried medicine. Among the folk medicinal plants, medicinal parts, or crude drugs, from eight species need fresh materials. The medicinal parts of 13 species must be dried materials, and the medicinal parts of 16 species can be fresh or dried material. There is a crossing situation of fresh and dry materials among the remaining three species, *Allium condensatum*, *Ephedra sinica*, and *Prunus humilis*. Specifically, the bulb skin of *Allium condensatum* should be dried to treat pregnant woman’s leg “  (bam)” and its fresh aerial part is used in treating toothache. The dried root of *Ephedra sinica* has an effect on treating rhinitis; the fresh aerial part works on relieving pain; the fresh or dried aerial part is used for treating pneumonia and joint pain. The dried roots of *Prunus humilis* are used to treat children with scabies; fresh roots bitten between the teeth or fresh or dried roots gargled in the mouth are both used for treating toothache. In the field of TMM and TCM, the medicinal parts of *Artemisia argyi*, *Sabina vulgaris*, and *Taraxacum mongolicum* are used fresh or dried; the medicinal part of *Cuscuta chinensis* is the fresh part; the fresh herbaceous stems of *Ephedra sinica* are used for making ointment in TMM; and the fresh material of *Cirsium maackii*, *Clinopodium chinense* subsp. *grandiflorum*, *Potentilla longifolia*, *Rhododendron micranthum*, and *Stellera chamaejasme* is used externally sometimes in TCM. Medicinal parts of *Glycyrrhiza uralensis* and *Prunus sibirica* are used fresh in TMM, but are dried in TCM. Medicinal parts of other species need to be dried.

#### Processing methods, folk recipe, and preparation

Through a simple process of sorting and washing, local people use the following processing methods: soaking, pulverizing, and mashing triturate, i. e., the fresh or dried root of *Glycyrrhiza uralensis* is soaked in milk before decocting, the fresh whole plant of *Cirsium maackii* is pulverized, and the fresh whole plant of *Oxytropis myriophylla* is mashed before using. In TMM and TCM, some special processing methods are used. For *Ephedra sinica* and *Glycyrrhiza uralensis*, the dried materials can be made as medicine directly or after baking with honey in TCM. In TMM, the dried materials of *Ephedra sinica* can be used as medicine directly or after roasted into carbon and the fresh materials can be made as a medical ointment. Dry seeds of *Prunus sibirica* can be made into medicine after heating up in boiling water or after stir-frying in TCM. Dried roots of *Scutellaria baicalensis* are cut in pieces and stir-fried with yellow rice wine in TCM. The root of *Stellera chamaejasme* can be made as medicine directly or after baking with vinegar in TCM.

Most crude materials can be used alone, like the simple recipe (or prescription) of TMM or TCM. Three types of folk medicine consist of two or more components, like a compound recipe of TMM or TCM. The dried roots of *Adenophora wulingshanica* and dried leaves of *Clinopodium chinense* subsp. *grandiflorum* are milled into powder and taken after mixing with water to treat cough, clearing heat and purging lung phlegm. The following seven different plant materials are covered in medicated bath, i.e., the fresh or dried whole plant of *Artemisia argyi*, the fresh or dried root of *Polygonum aviculare*, the fresh or dried aerial part of *Ephedra sinica*, the fresh or dried whole plant of *Artemisia frigida*, the fresh or dried branch of *Rhododendron micranthum*, the fresh or dried root of *Sanguisorba officinalis*, and the fresh or dried branch of *Sabina vulgaris.* In a folk medicated bath, the preparation method is decoction and mixture in TMM or TCM. The following folk medicine, like liniments in TMM or TCM, can be applied on the infected parts of the body. The dried whole plant of *Artemisia brachyloba* works on relieving pain and reducing swelling through the method of smoking the affected parts of the body, which is the same with smoke fumigant in TCM. The fresh aerial part of *Ephedra sinica* has the medicinal function of relieving pain after roasting, grinding, and mixing with water, which is the same with powders in TMM. In addition, the root of *Euphorbia esula* can be used after removing the root bark and central hard part and boiling it with sheep bone soup. The fresh or dried root of *Glycyrrhiza uralensis* should be soaked in cow milk and then decocted with water. Sheep bone and cow milk are local special products in the pastoral areas, which illustrates that local folk medicine is closely associated with the local conditions.

#### Internal and external use

According to the drug regimen, a medication can be classified for internal or external use. Shown in Table 5, 19 species are externally used (47.5%), and 17 species are internally used (42.5%) (Table 6). One of the external use methods is when the raw materials are used for direct padding, daubing, biting, and filling through a simple physical processing, e.g., pulverizing, cutting, rolling, or mashing. Another external use method is when the raw materials are used for smelling, washing, smoking, gargling, or bathing through a simple chemical processing, such as calcining or decocting. Decocting with water and taking medicine after mixing with water are the most common orally used methods. Externally used medicinal plants include *Cirsium vlassovianum* which is indirectly used for the treatment of rheumatism and joint pain by roasting silver acupuncture needles on a base of the papery part of the leaves. The most simple method for internal use is chewing, i.e., the fresh roots of *Erodium stephanianum* can be eaten after low fire roast, the dried seed of *Pinus tabuliformis* can be directly eaten after frying, and the fresh whole plant of *Sonchus arvensis* and *Urtica cannabina* can be eaten like a vegetable. This may be considered as a folk dietetic treatment. Tender stems and leaves of *Urtica cannabina* are also used as a folk dietotherapy by the Mongolians in Arhorchin Banner [23]. The four species of *Allium condensatum*, *Ephedra sinica*, *Stellera chamaejasme*, and *Urtica cannabina* can be used as external or internal medicine. Among them, we believe that smelling *Allium condensatum* and *Ephedra sinica* belongs to internal use because the special odor of roasting the bulb skin is drawn into the lungs during nasal inhalation.

**Table 6:** Internally and externally used method of folk medicine, TMM and TCM

Medicinal plants	Folk medicine	TMM	TCM
*Adenophora wulingshanica*	in	–	in-ex
*Allium condensatum*	in-ex	–	–
*Artemisia argyi*	ex	in	in-ex
*Artemisia brachyloba*	ex	in	in
*Artemisia frigida*	ex	in-ex	in-ex
*Cirsium maackii*	ex	–	in-ex
*Cirsium vlassovianum*	ex	–	in
*Clinopodium chinense* subsp. *grandiflorum*	in	–	in-ex
*Cuscuta chinensis*	ex	in	in
*Ephedra sinica*	in-ex	in-ex	in-ex
*Erodium stephanianum*	in	in	in
*Euphorbia esula*	ex	–	in-ex
*Glycyrrhiza uralensis*	in	in	in
*Incarvillea sinensis*	ex	in	in-ex
*Iris dichotoma*	ex	–	in-ex
*Leontopodium leontopodioides*	ex	in	iIn
*Oxytropis myriophylla*	ex	in	in-ex
*Pinus tabuliformis*	in	in	in-ex
*Plantago depressa*	in	in	in
*Polygonum aviculare*	ex	–	in-ex
*Potentilla longifolia*	in	in	in-ex
*Prunus humilis*	ex	–	in
*Prunus sibirica*	in	in	in
*Pulsatilla chinensis*	in	in	in
*Pyrus ussuriensis*	in	in	in
*Rhododendron micranthum*	ex	in-ex	in-ex
*Sabina vulgaris*	ex	in-ex	in-ex
*Sanguisorba officinalis*	ex	–	in-ex
*Saposhnikovia divaricata*	in	–	in
*Scutellaria baicalensis*	in	in	in-ex
*Scutellaria viscidula*	in	in	in
*Sonchus arvensis*	in	in	in-ex
*Sophora flavescens*	in	in	in-ex
*Sphaerophysa salsula*	ex	–	in
*Stellera chamaejasme*	in-ex	in-ex	in-ex
*Taraxacum mongolicum*	in	in	in-ex
*Thymus serpyllum* var. *asiaticus*	ex	–	in
*Tribulus terrestris*	in	in	in
*Urtica cannabina*	in-ex	in-ex	in-ex
*Xanthium mongolicum*	ex	–	–

*in* internal, *ex* external

Among the 26 species of folk medicinal plants which have been recorded in the literature of TMM, if we do not consider the differences of medicinal parts between folk medicine and TMM, 17 species can be internally and internally-externally used and the similarity rate reaches 65.38%. However, 6 species are externally and internally-externally used with a similarity rate of 23.08%. In terms of internal and external use, folk medicine and TMM are more similar in internal use. Comparing between the plant species of folk medicine and TCM, the internally used 20 species and the externally used 14 species reached a similarity rate of 52.63% and 36.84%, respectively. There is no difference between folk medicine and TCM. It can be seen that the differences between folk medicine and TMM are greater than differences between folk medicine and TCM in terms of internal and external use. Comparing between the 26 species of TMM and TCM, 11 species are internally used and 6 species are internally-externally used (the similarity rate of 65.38%). The other nine species are used internally in TMM and are used internally-externally in TCM. There is no instance where the opposite method of internal or external used between them. In TCM, 15 species are used both internally and externally, which shows the diversified characteristics of TCM in medicating methods.

### Functions and indication of folk medicine and their similarities of TMM and TCM

#### Functions and indication of folk medicine

It can be seen that local Mongol herdsmen understand the indications or functions of medicinal wild plants. The specific indications of 25 species of plants were offered by the local herdsmen. Another 4 species were only offered the functions simply, and another 11 species were offered the functions and indications. Thirty-six species of medicinal plants used by local herdsmen may cure 28 kinds of diseases. Cough, joint pain, toothache, and trauma are more common illnesses (Table 7).

**Table 7:** Indication and its frequency and related species

Diseases	Frequency	Number of related species
Cough	7	8
Joint pain	6	10
Toothache	4	4
Trauma	4	4
Diarrhea	2	2
Nose hemorrhage	2	2
Pneumonia	2	2
Rheumatism	2	2
Stomachache	2	2
Back leg pain	1	1
Body pain	1	1
Body shiver	1	1
Children scabies	1	1
Enteritis	1	1
Fatty liver	1	1
Gum swelling	1	1
Hemorrhoids	1	1
Headache	1	1
Hepatobiliary disease	1	1
Neuropathy	1	1
Renal edema	1	1
Rhinitis	1	1
Tracheitis	1	1
Vomit	1	1
Viper bites and analgesic effect	1	1
Children do not grow hair	1	1
Pregnant woman’s leg “ (bam)”	1	1
“ (ʃɑr)” disease	1	1
Total	50	55

Among them, the word “ (bam)” is derived from Tibetan, a special terminology of TMM, indicating “scorbutus” or “scurvy” in English. In TMM, “ (ʃɑr)” refers to “hotness”. The heat of the organs and spirit are determined by “ (ʃɑr)”. Too much “ (ʃɑr)” is revealed in a bitter taste in the mouth, sourness, or anxiety in mood and illness.

The functions of medicinal plants offered by local herdsmen included clearing heat-fire, detoxifying, reducing swelling, heat-clearing, reducing phlegm, thermal spa treatment, and dispelling coldness. Fifteen species of medicinal plants used by local herdsmen have 13 kinds of curative effects (Table 8).

**Table 8:** Function and its frequency and related species

Function	Frequency	Number of related species
Clearing heat-fire	4	4
Detoxifying	3	3
Reducing swelling	3	3
Heat-clearing	2	2
Reduce phlegm	2	2
Thermal spa treatment	2	2
Dispel coldness	1	2
Blood circulation	1	1
Clear away lung heat	1	1
Disinfect	1	1
Reducing inflammations	1	1
Relieving pain	1	1
Stopping hemorrhage	1	1
Total	22	23

#### Functions and indications compared between folk medicine with TMM and TCM

“Function” refers to the therapeutic effects of TMM or TCM. “Indication” refers to the types of diseases the functions of a medicine can address. There is an inherent relationship between function and indication. Generally, only one or two functions and indications of folk medicine were provided by local Mongol herdsmen. However, functions and indications of TMM and TCM have more diversity and systematic characteristics. More and more functions and indications have been recorded in TMM and TCM. In this paper, we discussed how one or two functions of folk medicine correspond to one or two diversified functions of TMM and TCM.

### Functions

In folk medicine, only the functions of *Cirsium maackii*, *Polygonum aviculare*, *Sonchus arvensis*, and *Sophora flavescens* were offered. Not only functions but also indications of another 11 species were offered. Among them, folk functions of 12 species can be found in diversified functions of TMM or TCM. The records of the folk functions of *Ephedra sinica*, *Polygonum aviculare*, and *Thymus serpyllum* var. *asiaticus* have not been found in TMM or TCM.

*Adenophora wulingshanica* works to “clear away lung heat and reduce phlegm” in folk medicine. In TCM, it has an effect on “heat-clearing, reducing phlegm, and detoxifying.” The function of “clear away lung heat” has an intrinsic relation with “heat-clearing” and “reduce phlegm,” which is the same between folk medicine and TCM.

*Artemisia argyi*, externally used for “thermal spa treatment,” can “dispel coldness and disinfect” in folk medicine, which has not been recorded in the TMM literature. In TCM, it can be internally-externally used and works on “warming channel for arresting bleeding, dissipate cold, and stop pain.” In fact, thermal spa treatment has the combined effect; the mechanism is from the external use of the physical (temperature and mechanical) and chemical action to promote the cerebral cortex gradually forming normal coordination activities, to inhibit and gradually replacing the pathological process of the disordered body, and to make chronic diseases be alleviated or cured. These treatments have a significant effect on skin, joint meridians, cardiovascular system, respiratory, gastrointestinal function, and trauma. The functions “thermal spa treatment,” “dispel coldness and disinfect,” and “warming channel for arresting bleeding, dissipate cold, and stop pain” represent an intrinsic relation between FMM and TCM.

*Artemisia brachyloba* can “reduce swelling” which corresponds to one of the six functions in TMM. In TCM, this plant has an effect on “heat-clearing and damp-drying” and “destroying parasites.” Literally, the functions have no direct relation to “reducing swelling.” However, there is an intrinsic relation between “heat-clearing” and “reducing swelling.” In order to reduce swelling, we can take the medicine for “heat-clearing.”

*Cirsium maackii* works on “reducing swelling” and “stopping hemorrhage” in folk medicine, which is not recorded in the TMM literature. In TCM, it has the medicinal functions of “cooling blood,” “stopping hemorrhage,” and “eliminating stasis and activating blood circulation and reducing swelling.” The functions of “reducing swelling” and “stopping hemorrhage” are the same in folk medicine and TCM.

*Clinopodium chinense* subsp. *grandiflorum* can be used for “heat-clearing” and to “reduce phlegm” in folk medicine. The function of “heat-clearing” is the same in TCM.

*Oxytropis myriophylla* has an impact on “reducing inflammations” in folk medicine. In TMM, it works on “destroying ‘ (nɪn)’ parasites,” “heat-clearing,” “removing ‘ (ʃɑr ʊs)’,” “healing wound,” “promoting granulation,” “stopping hemorrhage,” “reducing swelling,” and “relaxing the bowels.” The folk function of “reducing inflammations” has an intrinsic relationship with the functions of “heat-clearing” and “reducing swelling” in TMM. In TCM, it works on “heat-clearing and detoxifying,” “reducing swelling,” “dispelling rheumatism,” and “stopping hemorrhage.” The folk functions of “reducing inflammations” has an intrinsic relationship with the functions of “heat-clearing” and “reducing swelling” in TCM.

*Scutellaria baicalensis* can be used for “heat-clearing and detoxifying” in folk medicine and in TMM. In TCM, it has an effect on “heat-clearing and damp-drying,” “purging intense heat and detoxifying,” “stopping hemorrhage,” and “miscarriage prevention.” The functions of “heat-clearing” and “detoxifying” are the same between folk medicine and TCM.

*Scutellaria viscidula* is used for “heat-clearing and detoxifying” in folk medicine and in TMM. In TCM, it has an effect on “heat-clearing and damp-drying” and “detoxifying.” The function of “heat-clearing” and “detoxifying” is the same between folk medicine and TCM.

*Sonchus arvensis* can affect “blood circulation” and “heat-clearing and fire-purging” in folk medicine. The folk function of “blood circulation” is not found in TMM and TCM, but “heat-clearing and fire-purging” has an intrinsic relationship with the functions of “heat-clearing and detoxifying” in TMM and TCM.

*Sophora flavescens* works on “heat-clearing and detoxifying” in folk medicine. In TMM, it has the functions of “heat-transmission,” “promoting eruption,” and “removing ‘ (ʃɑr ʊs)’.” The folk function of “heat-clearing” has an intrinsic relationship with the functions of “heat-transmission” in TMM. In TCM, it has an effect on “heat-clearing and damp-drying,” “relieving itching,” “destroying parasites,” and “promoting urination.” The function of “heat-clearing” is the same between folk medicine and TCM.

*Stellera chamaejasme* has an impact on “reducing swelling” in folk medicine. In TMM, it works on “destroying ‘ (nɪn)’ parasites,” “removing retained water,” “reducing ‘ (ʧixɑ)’,” “removing putrefaction,” “reducing swelling,” and “promoting granulation.” The folk function of “reduce swelling” has an intrinsic relationship with the functions of “removing retained water,” and the same with “reducing swelling” in TMM. In TCM, it works on “removing retained water and reduce phlegm” and “clearing gore and destroying parasites.” The folk functions of “reducing swelling” have an intrinsic relationship with the functions of “removing retained water” and “clear gore” in TCM.

*Taraxacum mongolicum* is used for “heat-clearing and fire-purging” in folk medicine, and this function has an intrinsic relationship with the functions of “heat-clearing and detoxifying” in TMM and TCM.

### Indications

In 38 species of folk medicinal plants used in TMM or TCM, the indications of 36 species were offered by local herdsmen. Among them, the indications of 13 species in folk medicine correspond to 1 or 2 diversified indications in TMM. The indications of 16 species in folk medicine correspond to 1 or 2 diversified indications in TCM. An interesting case is that the indications for folk medicine are more similar with that of TCM than TMM. Sometimes, the indications for internal or external uses of the medicine are similar between folk medicine and TMM or TCM.

*Adenophora wulingshanica* is an orally used medicine for “cough” in folk medicine. It is not used in TMM. In TCM, it is an orally and externally used medicine. Five kinds of indications for this plant have been recorded. Among them, “cough with lung heat” is directly related to “cough.” Medicinally used parts, roots, are the same. In folk medicine, the dry roots of this plant and dry leaves of *Clinopodium chinense* subsp. *grandiflorum* are milled into powder and mixed and soaked in boiled water to take. However, in TCM, it is taken as a water-decocted solution of the roots. The difference between them is the oral administration method used.

*Artemisia brachyloba*, as an externally used medicine, has an indication of “ (ʃɑr) disease” (the body turns the color of yellow) in folk medicine. However, it is an orally used medicine in TMM and TCM. In TMM, the indications for the plant include brain pain, skin itching, carious pain, and five other diseases. According to the theory of TMM, “ (ʃɑr) disease” is a disease where its clinical features include fever, headache, heartburn, and sometimes spitting yellow acid water or diarrhea. Furthermore, the pain is centered in certain parts of the body. The eyes and skin turn the color of yellow, and the flavor of sweat becomes sour and stinks. Moreover, the body condition of the patient worsens at noon and midnight and at the time of digesting food in the thermal environment. Among them, one kind of “ (ʃɑr) disease” called “turning black  (ʃɑr)” shows symptoms: the skin of the patient gradually becomes yellow and green, and then a dark color, combined with itching skin, loss of hair, and rash [44]. We can see that the “ (ʃɑr) disease” in folk medicine has an intrinsic relationship with the “ (ʃɑr) disease” in TMM. The difference is internal and external treatment. In TCM, the indications include migraine, sore throat, and rheumatoid arthritis. We think that they have no direct relationship with “ (ʃɑr) disease”.

*Ephedra sinica* is used as an external medicine and also as an orally used medicine, works on “rhinitis (roots)” and “joint pain (aerial parts).” The indication is pneumonia (aerial part) in folk medicine. In TMM, as an orally used medicine, 14 kinds of indications of the plant (herbaceous stems) have been recorded. Among them, we think that the indications of TMM, “consumptive fever,” “ (ʃɑr) fever,” and “new and old fever,” may be related to pneumonia. As an externally used medicine, its herbaceous stem is used for a medicated bath in TMM. On this point of a medicated bath, usage of the plant is consistent between TMM and folk medicine. In TCM, eight indications of the plant (herbaceous stems) have been recorded. Among them, we think that only one indication of TCM, “dyspnea and cough,” may be related to pneumonia.

*Glycyrrhiza uralensis*, as an orally used medicine, has an impact on “cough” and “vomit” in folk medicine. In TMM, as an orally used medicine, nine indications for the plant have been recorded. Among them, we think that four indications of TMM, “cough with lung heat”, “tuberculosis”, and “dry mouth vomiting”, are directly or indirectly related to “cough” and “vomit”. In TCM, as an orally used medicine, 11 indications of the plant have been recorded. Among them, one indication of TCM, “cough”, is the same between folk medicine and TCM.

*Incarvillea sinensis*, as an externally used medicine, works on “toothache” and “joint pain” in folk medicine. In TMM, as an orally used medicine, eight indications for this plant have been recorded. Among them, we think that one indication of TMM, “ (ʃɑr ʊs) disease,” is related to “joint pain.” According to the theory of TMM, “ (ʃɑr ʊs) disease” is accompanied by loss of hair, joint-limb pain, difficulty to flexion and extension, backache, gum bleeding, etc. [22]. Basically, “ (ʃɑr ʊs) disease” leads to “joint pain.” The difference between them is internal and external treatment. In TCM, as an orally use medicine, two indications, “rheumatoid arthritis” and “bones and muscles spasm,” have been recorded. Basically, “rheumatoid arthritis” leads to “joint pain.” As an externally used medicine, indications for the plant including eczema, aphthosis, and carbuncle are in TCM.

*Iris dichotoma* is an externally used medicine, which has an effect on “toothache and gum swelling” in folk medicine. It is not used in TMM. In TCM, nine indications of this plant have been recorded. Among them, we think that the indication, “swelling and aching of gum,” is basically the same as “toothache and gum swelling.” However, it is an orally used medicine in TCM. As an externally used medicine, in TCM, it is decocted in water and then cleaned, or the pounded raw material is applied in the affected part of the body. In folk medicine, the root of this plant is cut into pieces and bitten between the upper and lower teeth.

*Oxytropis myriophylla* is an externally used medicine that works on “nose hemorrhage” and “trauma” in folk medicine. In TMM, as an orally used medicine, 16 kinds of indications for this plant have been recorded. Among them, the indication of “nose hemorrhage” and “trauma” are consistent between folk medicine and TMM. However, a very interesting case is that the same indication appears not only as an externally used medicine, but also as an orally used medicine. In TCM, as an external and orally used medicine, six indications for this plant have been recorded. Among them, the indication, “trauma,” is consistent between folk medicine and TCM. Another indication in TCM, “various hemorrhage,” has a direct relation with that of “nose hemorrhage” in folk medicine. As an externally used medicine, in TCM, the pounded raw material is applied to cure the affected part. This is similar to the folk medicine method of using the fresh whole plant mashed and then daubed on the infected part to treat “nose hemorrhage” and “trauma.”

*Plantago depressa*, as an orally used medicine, has an impact on “stomachache” and “diarrhea” in folk medicine. In TMM, as an orally used medicine, six indications for this plant have been recorded. Among them, “diarrhea” and “intestine stabbing pain” are directly or indirectly related to “stomachache” and “diarrhea” in folk medicine. In folk medicine, a water-decocted solution of fresh or dried roots of this plant is taken. In TMM, the dried seeds of this plant are used for a pill and powder medicine. The difference between them is the medicinally used parts and use method. The records of the folk indication are not found in TCM.

*Potentilla longifolia*, as an orally used medicine, works on “enteritis” in folk medicine. In TMM, as an orally used medicine, four indications for this plant have been recorded. Among them, the folk indication is not found in TMM. In TCM, as an orally and external orally used medicine, ten indications for this plant have been recorded including “acute enteritis” which is closely related to “enteritis” in folk medicine.

*Prunus sibirica* is an orally used medicine used for “cough,” “pneumonia,” and “tracheitis” in folk medicine. In TMM, as an orally used medicine, six indications for this plant have been recorded. Among them, “cough” is the same with folk medicine and TMM. Another indication in TMM, “asthma,” has a direct relationship with that of “pneumonia” and “tracheitis” in folk medicine. In TCM, as an orally used medicine, indications for “cough and asthma,” “excessive phlegm,” and “intestinal dry constipation” have been recorded. We think that the indication, “cough and asthma” and “excessive phlegm,” has a direct correlation with the indication of the folk medicine, “cough,” “pneumonia,” and “tracheitis.”

*Rhododendron micranthum*, as an externally used medicine, has an effect on “joint pain” in folk medicine. In TMM, as an orally used medicine, 13 indications for this plant have been recorded. Among them, we think that the indication in TMM, “limbs stiff flexion,” is related to “joint pain” in folk medicine. This plant is also used externally in TMM. Its leaves or flowering branches can be used in a medicated bath. The usage of a medicated bath with this plant is consistent between TMM and folk medicine. In TCM, as an orally used medicine, six indications for this plant have been recorded. Among them, “rheumatic arthralgia” and “joint pain after childbirth” are closely related to “joint pain” in folk medicine. This plant is also used externally in TCM, and it is aimed to treat “cataclasis” and “sores.” The usual treatment is that the pounded fresh materials are applied to the affected part of the body or the affected part is cleaned with the decocted water.

*Sabina vulgaris*, as an externally used medicine, has an impact on “joint pain” in folk medicine. In TMM, as an orally used medicine, nine kinds of indications for the plant have been recorded. Among them, we think that the indication, “ (ʃɑr ʊs) disease,” is related to “joint pain” in folk medicine because “ (ʃɑr ʊs) disease” leads to “joint pain.” This plant is also used externally in TMM. Its branches and leaves are used in a medicated bath. The usage of a medicated bath with this plant is consistent between TMM and folk medicine. In TCM, as an orally used medicine, five indications for this plant have been recorded. Among them, the indication, “rheumatoid arthritis,” is closely related to “joint pain” in folk medicine. This plant is also used externally in TCM. The usual treatment is that the affected part is cleaned by the decocted water or smoke the affected part after baking.

*Saposhnikovia divaricata*, as an orally used medicine, works on “rheumatism” and “joint pain” in folk medicine. It is not used in TMM. In TCM, as an orally used medicine, seven indications for this plant have been recorded. Among them, “rheumatism and joint pain” is the same between folk medicine and TCM.

*Scutellaria baicalensis*, as an orally used medicine, has an indication of “cough” in folk medicine. In TMM, as an orally used medicine, four indications for this plant have been recorded. Among them, “cough with lung heat” is closely related to “cough.” In TCM, as an orally and externally used medicine, nine indications for this plant have been recorded. Among them, “cough with lung heat” is the same as in TMM and related to “cough” in folk medicine.

*Scutellaria viscidula*, as an orally used medicine, has an effect in “cough” in folk medicine. In TMM, as an orally used medicine, four indications for this plant have been recorded. The indications for this plant are totally the same as the indications for *Scutellaria baicalensis* in TMM. Among them, “cough with lung heat” is directly related to “cough.” In TCM, as an orally used medicine, six indications for this plant have been recorded. The indication of “cough with lung heat” is the same in TMM and related to “cough” in folk medicine. The difference between them is the orally used method. In folk medicine and TCM, a water-decocted solution is taken, but in TMM, it is used for a pill and powder medicine.

*Taraxacum mongolicum*, as an orally used medicine, has an impact on “hepatobiliary disease” and “fatty liver” in folk medicine. In TMM, as an orally used medicine, 12 indications for this plant have been recorded. Among them, we think that only one indication of “jaundice” may be related to “hepatobiliary disease.” According to the theory of TMM, “jaundice” is “ (nɪn)” infection to the human body, through blood circulation to the liver, and blood diffusion “ (ʃɑr)” heat led to the normal mechanism of liver and gallbladder. The main manifestations are the eyes and body skin turn the color of yellow, urine and excrement turn yellow, vomiting, and so on [43]. In TCM, as an orally and externally used medicine, 16 indications have been recorded. Among them, the indication of “acute icteric hepatitis” and “cholecystitis” may be related to “hepatobiliary disease” and “fatty liver” in folk medicine. According to the theory of “acute icteric hepatitis,” this is a kind of acute viral hepatitis. The clinical manifestations are acute loss of appetite, distaste for oil, fatigue, upper abdomen discomfort, liver pain, nausea, vomiting, and, for some patients, chills, fever, and then urine color deepening, sclera, and skin jaundice [45].

*Urtica cannabina*, as an orally and externally used medicine, has an effect on “joint pain” and “viper bites” and an “analgesic effect” in folk medicine. In TMM, as an orally used medicine, although five indications for this plant have been recorded, there is no indication related to “joint pain.” In TCM, as an orally used medicine, seven indications for this plant have been recorded. Among them, the indication “rheumatoid arthritis” is closely related to “joint pain” in folk medicine. This plant is also used externally in TMM and TCM. The indication of “bites caused by insects and snakes” in TMM and TCM is closely related with “viper bites and analgesic effect” in folk medicine. The usual treatment is the usage of decocted water to clean the affected part in TCM or the application of the pounded material in the affected area in TMM and TCM.

## Conclusion

There are not many wild plant species used for medicine by the Mongol herdsmen in the Bairin Area. However, there are 14 species that have not been recorded in TMM and 2 species that have not been recorded in TCM. Folk knowledge may have a certain reference value to search for wild plant species. The local Mongol herdsmen frequently use herbaceous plants, especially the perennial herbs, for medicine. This accords with the flora characteristics of temperate grassland region. The roots, whole plant, and aerial parts are the more frequently used. Compared with TMM and TCM, traditional folk medicine can provide useful information to find new medicinal parts of wild plant species.

The paper analyzed four aspects of folk methods for preparing medicine including fresh and dried medicine, processing methods, folk recipes and preparation, and orally and externally used medicine. On the whole, the fresh medicines were commonly used, the boiling method for processing is the most commonly used method, most crude materials are used alone, and externally used medicines are more common than those used orally. Folk medicinal baths may be regarded as compound recipe combined, and seven (two kinds of medicine) plant species have been related. One kind of medicine is including Artemisia argyi and Polygonum aviculare, another kind of medicine is including Artemisia frigida, Ephedra sinica, Rhododendron micranthum, Sanguisorba officinalis, Sabina vulgaris, to bathing.

For indication and function, the local Mongol herdsmen fully understand the function and indication of folk medicines. The functions offered by local herdsmen included clearing heat-fire, detoxifying, reducing swelling, heat-clearing, reducing phlegm, thermal spa treatment, and dispelling coldness. The folk medicines may cure 28 kinds of diseases. Cough, joint pain, toothache, and trauma are the more common illness. The local herdsmen understand more about indications than functions of folk medicine.

## Abbreviations

FMM: Folk Mongolian Medicine
MFM: Mongolian Folk Medicine
TCM: Traditional Chinese Medicine
TMM: Traditional Mongolian Medicine
